# Positive association of nap duration with risk of non-alcoholic fatty liver disease in an occupational population in Guangdong Province, China: a cross-sectional study

**DOI:** 10.1186/s12876-022-02246-5

**Published:** 2022-04-12

**Authors:** Chang Hong, Chengkai Wu, Pengcheng Ma, Hao Cui, Liya Chen, Ruining Li, Qimei Li, Lin Zeng, Shengwu Liao, Lushan Xiao, Li Liu, Wenyuan Li

**Affiliations:** 1 Big Data Center, Nanfang Hospital, Southern Medical University, Guangzhou, 510515 China; 2 Department of Infectious Diseases, Nanfang Hospital, Southern Medical University, Guangzhou, 510515 China; 3 Department of Radiation Oncology, Nanfang Hospital, Southern Medical University, Guangzhou, 510515 China; 4 Department of Medical Quality Management, Nanfang Hospital, Southern Medical University, Guangzhou, 510515 China; 5 Hospital Office, Nanfang Hospital, Southern Medical University, Guangzhou, 510515 China

**Keywords:** NAFLD, Health, Nap, Risk

## Abstract

**Background:**

A lack of sleep or disorder in sleep–wake cycles has been associated with metabolic impairments. However, few studies have investigated the association between daytime napping duration and the risk of non-alcoholic fatty liver disease. This study aimed to investigate the association of daytime napping duration with the risk of non-alcoholic fatty liver disease in a Chinese population.

**Methods:**

This cross-sectional study analyzed data from the Health Management Center of Nanfang Hospital, Guangdong Province. A total of 3363 participants aged 20–79 years were recruited and admitted from January 20, 2018, to October 16, 2020. Non-alcoholic fatty liver disease was diagnosed using abdominal ultrasonography. The outcome was the association between daytime sleep duration and the risk of non-alcoholic fatty liver disease.

**Results:**

Compared with non-nappers, long daytime nappers (≥ 60 min) were associated with a higher risk of non-alcoholic fatty liver disease in the crude model (odds ratio 2.138; 95% confidence interval 1.88–2.61, *P* < 0.05) and in the multivariable adjustment model (odds ratio 2.211; 95% confidence interval 1.042–4.690, *P* < 0.05) after adjusting for demographic, educational, and metabolic risk factors. The association was moderately enhanced with additional adjustments for night sleep duration and socioeconomic or other factors (odds ratio 2.253; 95% confidence interval 1.061–4.786, *P* = 0.035).

**Conclusion:**

In this cross-sectional study, daytime napping duration of ≥ 60 min was positively associated with the risk of non-alcoholic fatty liver disease in an occupational population of Guangdong Province after multivariable adjustment.

**Supplementary Information:**

The online version contains supplementary material available at 10.1186/s12876-022-02246-5.

## Background

With changes in dietary and lifestyle habits, non-alcoholic fatty liver disease (NAFLD) has become the most prevalent liver disease in both developed and developing countries [1, 2], with a current global incidence of approximately 25% [2, 3]. NAFLD poses a significant threat to people’s health and imposes a great burden on the social economy. Moreover, NAFLD is currently the most important emerging cause of hepatocellular carcinoma (HCC) in developed countries [4, 5]. Approximately 20%–30% of individuals with NAFLD develop non-alcoholic steatohepatitis (NASH), which progresses into cirrhosis in 10%–20% of cases [6]. As a result, more attention should be paid to the vulnerable group to reduce the risk of NAFLD and its associated complications and mortality. NAFLD is a multisystemic disease with a histologic spectrum that ranges from macrovesicular steatosis to hepatic inflammation and fibrosis indicative of NASH [2, 4]. Liver biopsy remains the gold standard for the clinical diagnosis of NAFLD [7]. In clinical practice, the initial diagnosis of NAFLD is usually radiological and based on the presence of fat within the liver that involves at least 5% of the liver weight [8, 9]. Currently, the most used imaging technique is abdominal ultrasonography, which has several advantages including good accuracy and relative ease of performance [8].

Optimal sleep duration is necessary for maintaining normal physical, cognitive, and psychological functions. Many people have a habit of diurnal napping, which is widely recommended as a safe and non-invasive intervention to counteract the negative effects of partial sleep deprivation. Souabni et al. reported that diurnal napping improves short-term memory, attention, reaction time, repeated-sprint, endurance, and specific skill performance, with greater improvement following longer naps [10]. However, a long napping duration may be detrimental to one’s health. Yin et al. confirmed that daytime napping duration is positively associated with the risk of hyperuricemia in a Chinese population [11] and that there is a U-shaped relationship between diurnal naps and diabetes [12].

Therefore, in this cohort study, we aimed to investigate the association between daytime napping duration and the risk of developing NAFLD among the working-age population of Guangdong Province using physical examination data from Nanfang Hospital.

## Methods

### Study design and population

**Fig. 1:**
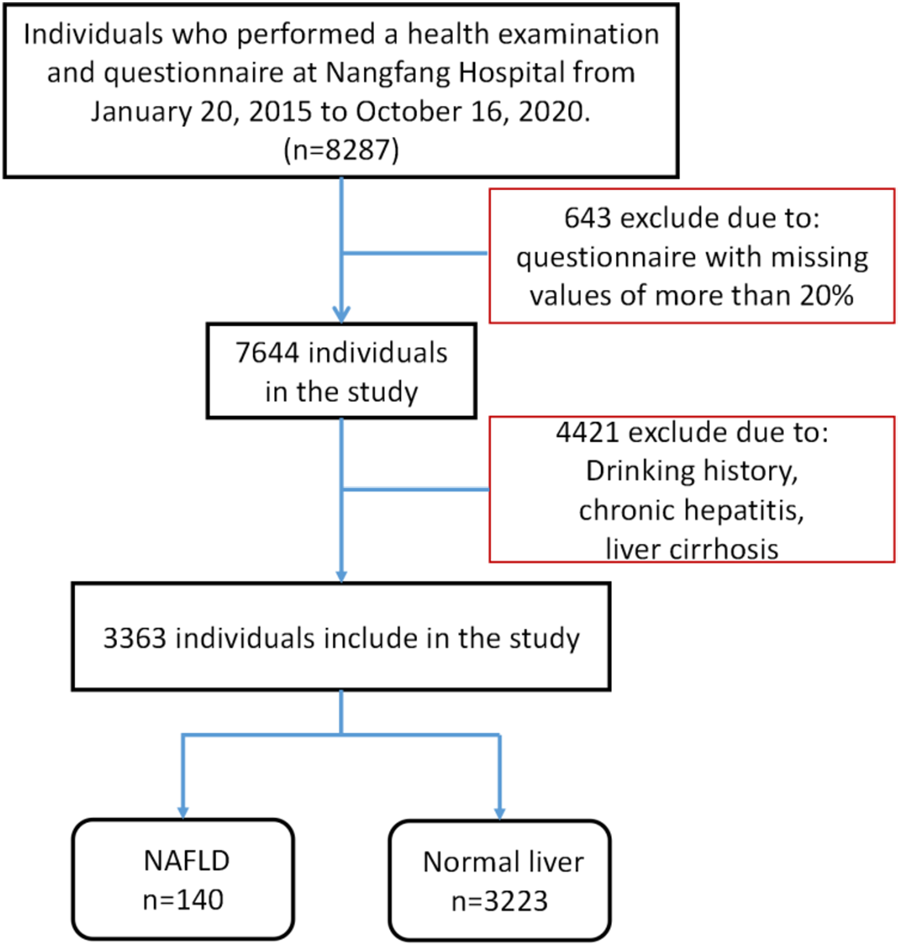
Study flowchart of participant selection

This cohort study used physical examination data from the Health Management Center of Nanfang Hospital. This regional cross-sectional study included 8,295 respondents. Questionnaire characteristics with missing values of more than 20% were eliminated; consequently, 7,644 questionnaires were included. We investigated whether participant had fatty liver via past medical history. Given the lack of records on alcohol consumption, 4,251 participants with a history of current or previous alcohol intake and 30 participants with chronic hepatitis or liver cirrhosis were excluded. Finally, a total of 3,363 individuals aged 20–79 years were included in the study, and the study flowchart is shown in Fig. 1. All study participants provided informed consent, and the study design was approved by the appropriate ethics review board of Nanfang Hospital.

### Daytime napping and nighttime sleep duration

Participants were asked about the duration of napping after lunch and the total night sleep duration. Daytime naps are usually brief periods of sleep lasting from a few minutes to a few hours [13]. Such naps may vary in frequency from the occasional nap to planned periods of rest up to several times each day in habitual nappers. Daytime napping duration was categorized as none, < 30 min, 30–60 min, and ≥ 60 min per day. Nighttime sleep duration was classified into ≤ 7 h and > 7 h per day.

### Covariates

Information on sociodemographic and lifestyle variables, including age, sex, educational level (below high school, college, bachelor’s degree, and above), smoking status (never, smoking, quit smoking for more than 6 months, and passive smoking), and physical activity (< 1 time and ≥ 1 time per week), was collected through a questionnaire survey. Total cholesterol, triglyceride, low-density lipoprotein cholesterol (LDL-c), and high-density lipoprotein cholesterol (HDL-c) levels were measured using an enzymatic colorimetric test.

### Statistical analysis

Baseline characteristics are presented as frequency with percentages for categorical variables and mean with standard deviation for continuous variables. Differences between the NAFLD and non-NAFLD groups were compared using analysis of variance for continuous variables and chi-square tests for categorical variables.

Subsequently, logistic regression analyses were performed to evaluate the association of daytime napping duration with the risk of NAFLD. Model 1 was the crude model. Model 2 was further adjusted for age (years), sex (men and women), and body mass index (BMI). Model 3 was additionally adjusted for educational level, annual family income, physical exercise, smoking status, daily sedentary duration, staying up late at night, and sleeping pill use. Furthermore, Model 4 was adjusted for hypertension, diabetes, coronary disease, and serum levels of total cholesterol, triglyceride, HDL-c, and LDL-c. To account for the mediating role of nocturnal sleep duration and dietary habit, Model 5 included these variables. All statistical analyses were performed with IBM SPSS Statistics for Windows version 25.0 (IBM Corp., Armonk, NY, USA).

## Results

A total of 3363 participants were included in this study, and 140 individuals were diagnosed with NAFLD via ultrasonography, which accounted for approximately 4.3% of the study population. Among the 3223 working-aged individuals without NAFLD, 63.7% were men, and the mean age was 33 ± 8 years. In addition, 391 individuals reported no daytime napping and 833 had a daytime napping duration of ≥ 60 min every day. Findings of the comparison of the characteristics between the non-NAFLD and NAFLD groups are shown in Table 1. In general, participants with NAFLD were more likely to have longer daytime napping duration (≥ 30 min per day). Moreover, those with NAFLD tended to take sleeping pills and have a longer daily sedentary duration.

**Table 1:** Comparison of baseline characteristics according to NAFLD

Characteristics	Without NAFLD (n = 3223)	With NAFLD (n = 140)	*P* value
Age, year	33 ± 8	37 ± 11	< 0.001
Male, n(%)	2053 (63.7%)	86 (61.4%)	0.585
BMI, kg/m^2^	22.3 ± 3.3	21.9 ± 3.2	0.174
High school above, n(%)	2632 (81.7%)	109 (77.9%)	0.323
Annual family personal income (thousand yuan)			0.601
< 20	317 (9.8%)	15 (10.7%)	
20–40	236 (7.3%)	12 (8.6%)	
40–60	298 (9.2%)	16 (11.4%)	
60–80	473 (14.7%)	16 (11.4%)	
Physical activity ≥ 1 time/week, n (%)	1230 (38.2%)	65 (46.4%)	0.06
Sleeping pills using	193 (6.0)	16 (11.4%)	0.009
Nocturnal sleeping duration/h, n(%)			0.058
≤ 7 h	2154 (66.8%)	103 (73.6%)	
> 7 h	1069 (33.2%)	37 (26.4%)	
Daytime dapping duration/min, n(%)			0.043
0	391 (12.1%)	9 (6.4%)	
< 30 min	346 (10.8%)	11 (7.9%)	
30-60 min	1653 (51.3%)	79 (56.4%)	
≥ 60 min	833 (25.8%)	41 (29.3%)	
Smoking, n(%)			0.896
Never	2984 (92.6%)	126 (90.0%)	
Current	141 (4.4%)	11 (7.9%)	
Quitted	32 (1.0%)	2 (1.4%)	
Passive	66 (2.0%)	1 (0.7%)	
Often staying up late	1751 (54.3%)	71 (50.7%)	0.377
Sedentary duration/(h per day)			0.387
> 8	703 (21.8%)	36 (25.7%)	
Coronary disease, n (%)	22 (0.7%)	0	0.999
Diabetes, n (%)	7 (0.2%)	0	0.742
Hypertension, n (%)	64 (2.0%)	8 (5.7%)	0.007
LDL-C	3.1 (0.7)	3.2 (0.6)	0.075
Triglycerides	1.2 (0.9)	1.2 (0.9)	0.672
HDL-C	1.3 (0.2)	1.4 (0.3)	0.028
Total cholesterol	4.8 (0.9)	5.0 (0.8)	0.014

BMI, body mass index; LDL-C, low-density lipoprotein cholesterol; HDL-C, high-density lipoprotein cholesterol

The characteristics among the groups stratified by daytime napping duration are shown in Table 2. Eighty (77%) participants with NAFLD had a daytime napping duration more than 30 min per day. Interestingly, compared with non-nappers, participants with ≥ 60 min of daytime napping were more likely to have a higher educational level, to be diagnosed with hypertension, and less likely to have a long daily sedentary duration and stay up late. Importantly, we observed an increasing trend in the difference of the incidence of NAFLD across the four daytime napping durations (2.3% in non-nappers, 2.6% in participants with < 30 min of daytime napping, 4.5% in those with 30–60 min of daytime napping, and 4.7% in those with ≥ 60 min of daytime napping).

**Table 2:** Comparison of baseline characteristics according to different daytime napping duration

Group(n)	Daytime napping duration			
	0 min (400)	1-29 min (357)	30-59 min (1732)	≥ 60 min (874)
Age, year				
≤ 30	202 (50.5%)	139 (38.9%)	624 (36.0%)	384 (43.9%)
30–40	131 (32.8%)	145 (40.7%)	702 (40.5%)	342 (39.1%)
40–50	53 (13.3%)	54 (15.1%)	314 (18.1%)	110 (12.6%)
> 50	14 (3.5%)	19 (5.3%)	92 (5.4%)	38 (4.4%)
Male, n (%)	278 (69.5%)	250 (70.0%)	1114 (64.3%)	497 (56.9%)
BMI, kg/m^2^				
< 18.5	44 (11.0%)	44 (12.3%)	163 (9.4%)	95 (10.9%)
18.5–23.9	252 (63.0%)	232 (65.0%)	1053 (60.8%)	509 (58.2%)
≥ 24	104 (26.0%)	81 (22.7%)	516 (29.8%)	270 (30.9%)
High school above, n(%)	256 (64.0%)	343 (96.1%)	1413 (81.6%)	729 (83.4%)
Annual family personal income( thousand yuan/person)				
< 20	50 (12.5%)	47 (13.2%)	163 (9.4%)	76 (8.7%)
20–40	45 (11.3%)	25 (7.0%)	123 (7.1%)	59 (6.8%)
40–60	47 (11.7%)	29 (8.1%)	156 (9.0%)	86 (9.8%)
60–80	72 (18.0%)	48 (13.4%)	239 (13.8%)	134 (15.3%)
Physical activity ≥ 1time/week, n (%)	124 (31.0%)	129 (36.1%)	669 (38.6%)	376 (43.0%)
Sleeping pills using, n(%)	26 (6.5%)	26 (7.3%)	103 (5.9%)	54 (6.2%)
Nocturnal sleep duration > 7 h, n(%)	136 (34.0%)	95 (26.6%)	535 (30.9%)	340 (38.9%)
Non-smoker, n (%)	372 (93.0%)	330 (92.4%)	1604 (92.6%)	803 (91.9%)
Often staying up late	218 (54.5%)	195 (54.6%)	970 (56.0%)	449 (51.4%)
Sedentary duration/ (h per day)				
> 8	80 (20.0%)	100 (28.0%)	407 (23.5%)	156 (17.8%)
Coronary disease, n (%)	3 (0.8%)	1 (0.3%)	13 (0.7%)	5 (0.6%)
Diabetes, n (%)	0	0	7 (0.4%)	0
Hypertension, n (%)	6 (1.5%)	5 (1.4%)	40 (2.3%)	21 (2.4%)
Total cholesterol	4.8 (0.9)	4.8 (0.9)	4.8 (0.9)	4.8 (0.8)
LDL-C	3.0 (0.7)	3.0 (0.7)	3.1 (0.7)	3.1 (0.6)
Triglycerides	1.2 (0.8)	1.2 (0.9)	1.2 (0.9)	1.2 (1.0)
HDL-C	1.4 (0.3)	1.4 (0.3)	1.4 (0.2)	1.4 (0.2)
**Outcomes**				
NAFLD	9 (2.3%)	11 (3.1%)	79 (4.5%)	41 (4.7%)

BMI, body mass index; LDL-C, low-density lipoprotein cholesterol; HDL-C, high-density lipoprotein cholesterol

**Fig. 2:**
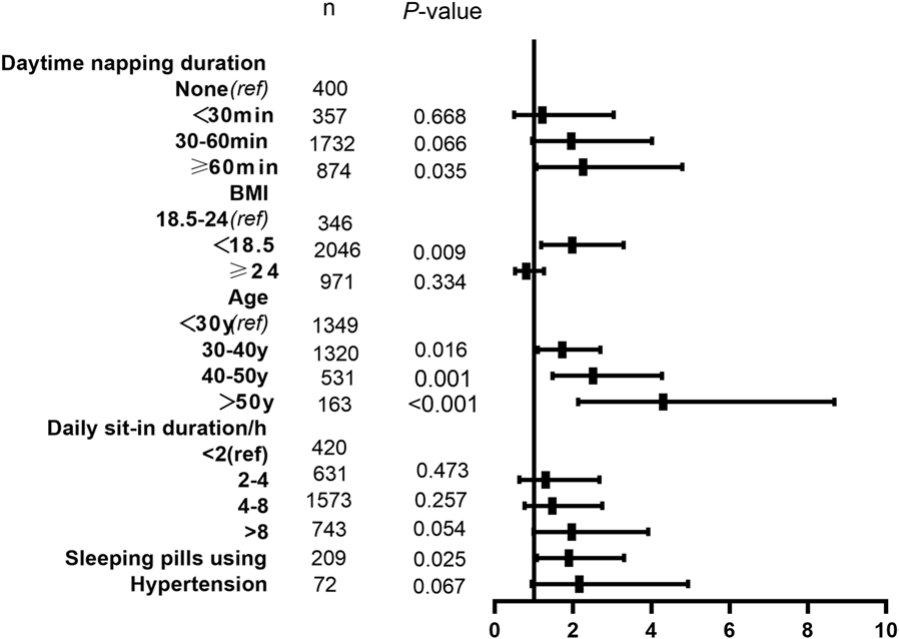
Forest plot shows OR (95% CI) of part of variables for NAFLD

To explore the association of daytime napping duration with the risk of NAFLD, five models, including different mediators, were created using logistic regression (Table 3 and Additional file 1). Model 1, which was also called the crude model, included only one variate, i.e., daytime napping duration. Compared with non-nappers, those with ≥ 60 min of daytime napping duration had a more than two-fold (odds ratio [OR] 2.138; 95% confidence interval [CI] 1.029–4.443) increased risk of NAFLD in the crude model with statistical significance. To rule out interference from other factors, the association between daytime napping and the risk of NAFLD was further evaluated, and an association was observed after controlling for other confounders. As shown in Table 3, the effect estimate was quite attenuated after introducing the mediators of age, sex, and BMI in Model 2 (OR 2.096; 95% CI 1.002–4.381). Especially, the ORs are increasing stepwise in the models (Table 3), and several variates, especially those that were found to be significant after further including demographic or lifestyle factors and clinical or biochemical variates in Model 5, are shown in Fig. 2. In addition, < 30 min of daytime napping duration was not significantly associated with the risk of NAFLD compared to the reference group in both crude and adjusted models. In the crude model, no significant association was observed between daily napping duration and the risk of NAFLD. However, a significant association was found after the inclusion of the variates. Additionally, < 30 min of daytime napping duration was not significantly related to the risk of NAFLD compared to the reference group in both crude and adjusted models. Consequently, we observed that daytime napping duration was positively associated with the risk of NAFLD in this working-aged cohort in Guangdong Province.

**Table 3:** Association between daytime napping duration and NAFLD

	Model 1	Model 2	Model 3	Model 4	Model 5
0	1(ref)	1(ref)	1(ref)	1(ref)	1(ref)
0-30 min	1.381	1.255	1.272	1.233	1.221
30-60 min	2.076*	1.888	1.920	1.956	1.959
≥ 60 min	2.138*	2.096*	2.155*	2.211*	2.253*

Model 1: crude model

Model 2: adjusted for age (years), sex (men and women) and body mass index (BMI)

Model 3: adjusted for age (years), sex (men and women), body mass index, education level, annual income per capita of the family, physical exercise, smoking, daily sedentary time, staying-up and sleeping pill use

Model 4: adjusted for age (years), sex (men and women), body mass index, education level, annual income per capita of the family, physical exercise, smoking, daily sedentary time, staying-up, sleeping pill use, total cholesterol, triglyceride, low-density lipoprotein cholesterol, high-density lipoprotein cholesterol, coronary heart disease, diabetes and hypertension

Model 5: model 4 plus adjusted for nocturnal sleep duration

* represents *P* value < 0.05

## Discussion

To the best of our knowledge, this is the first study to demonstrate that long daytime napping was positively associated with the risk of NAFLD, and this association remained significant after adjustment for sex, BMI, and other confounders. This excess risk appears to be independent of several socioeconomic factors and hypertension. The study participants are of working age, which is a relatively young cohort living in Guangdong Province. Therefore, the incidence rates of diabetes, coronary disease, overweight, and hypertension or their complications were lower than those of the total population. In this study, participants with hypertension are more likely to have a higher risk of NAFLD. Consistently, among middle-aged and older Chinese adults, individuals with longer daytime napping duration were associated with a significantly higher risk for hypertension compared to non-nappers, whereas reduced napping duration may confer a benefit for hypertension prevention [14]. Some studies have shown that high BMI, specifically > 30 kg/m^2^, is a risk factor for NAFLD [15, 16], but in this study, there was no statistical difference in the increased risk of NAFLD among participants with high BMI, which may account for the small proportion of obese people in our population. However, in our study, we found that lower BMI is risky for NAFLD. BMI is an index based in the weight and height, and one’s weight compose of bone, muscle, water and fat. Hence, a low or normal BMI does not mean you have less visceral fat, since fat is the least dense part of the body [17]. In our study, more than half of people with lower BMI exercise less than 1 time per week. Lack of physical activity, increase of waist hip rate, dysfunction of lipid metabolism or other reasons may explain this finding.

This study demonstrated a dose–response relationship between daytime napping duration and NAFLD. Although the exact mechanism underlying the association between daytime napping duration and the risk of NAFLD remains unknown, several theories may explain this relationship. First, long daytime napping duration could elevate the level of cortisol in the blood [18, 19], which might result in insulin resistance and disordered glucose and lipid metabolism [20]. Cortisol can promote the accumulation of lipids and visceral fat, including in the liver [21]. Second, a long napping duration could activate the sympathetic nervous system, resulting in an imbalance of sympathetic vagal balance and activation of the renin–angiotensin–aldosterone system [22, 23]. Consequently, the ability of pancreatic β-cells to secrete insulin is suppressed [12, 24, 25], which could lead to metabolic disturbances of glucose and lipids in the body. Third, obstructive sleep apnea is a known risk factor for NAFLD [26–28]. Individuals with obstructive sleep apnea who also have long daytime napping durations tend to be at a higher risk of obstructive sleep apnea. Fourth, people who take longer naps have lower levels of leptin [29, 30], which is secreted by lipocytes and regulates food intake and lipid metabolism by affecting the central nervous system. [31] In general, the downregulation of leptin in the body could promote appetite and consequently excessive energy intake, causing the accumulation of lipids and energy in the body and elevating the risk of NAFLD [32]. In addition, high levels of leptin could upregulate tumor growth factor-β [33], which is positively related to the risk of NAFLD [1]. In addition, an elevated leptin level may be a risk factor for insulin resistance. Last but not least, long daytime napping duration was shown to be related to high levels of pro-inflammatory cytokines in the body, such as interleukin-6 and C-reactive protein [34, 35], which are known risk factors for NAFLD development.

NAFLD is rapidly becoming the leading cause of HCC worldwide, and with the declining incidence of virus-related HCC, it is almost inevitable that NAFLD-related HCC will be the predominant etiology of HCC in many countries by 2030 [6, 36]. Importantly, many risk factors for the development of NAFLD are also risk factors for the onset of HCC. Therefore, effective screening of at-risk individuals, risk-based interventions, and surveillance could reduce the onset of NAFLD, identify patients with pre-cirrhotic NAFLD, and ultimately reduce the occurrence of NAFLD-related HCC and HCC-related mortality. In our study, long daytime napping duration increased the risk of developing NAFLD, along with age, BMI, and hypertension. These results highlight the potential value of lifestyle interventions focused on exercise and healthy eating habits.

However, there are still several limitations of our study. Firstly, the amount of alcohol consumed by the participants was unknown; thus, individual drinkers were excluded, leading to a reduction in the sample size analyzed. Second, most of the data were gathered using questionnaires, which may result in recall bias. Third, this is a cross-sectional study, which precludes us from determining the longitudinal impact of napping on NAFLD. Finally, although we have adjusted for several potential factors, some inaccurately measured or unknown factors could lead to residual bias.

## Conclusion

In this cross-sectional study, daytime napping duration of ≥ 60 min was positively associated with the risk of NAFLD in an occupational population of Guangdong Province after adjustment for socioeconomic factors, daily lifestyle, and other factors. This study demonstrates the potential negative effect of excessively long daily napping on NAFLD. Nevertheless, these results should be interpreted with caution because of the quality of the evidence, risk of bias, and limited evidence of napping interventions.

## Supplementary Information

**Additional file 1. **Adjusted odds ratios (95% CI) for risk of non-alcoholic fatty liver disease using logistic regression. The table showed the odds ratios (95% CI) for risk of non-alcoholic fatty liver disease in the univariate and multivariate logistic regression models. And age, BMI, hypertension, sleeping pills using and napping over 60min per day is probably the independent risk for developing NAFLD.

## Data Availability

The data supporting the findings of this study are available from the corresponding author upon reasonable request.

## Abbreviations

BMI: Body mass index
CI: 95% Confidence interval
HCC: Hepatocellular carcinoma
HDL-c: High-density lipoprotein cholesterol
LDL-c: Low-density lipoprotein cholesterol
NAFLD: Non-alcoholic fatty liver disease
NASH: Non-alcoholic steatohepatitis
OR: Odds ratio

## References

[1] Fontes-Cal TCM, Mattos RT, Medeiros NI (2021). Crosstalk between plasma cytokines, inflammation, and liver damage as a new strategy to monitoring NAFLD progression. Front Immunol.

[2] Huang DQ, El-Serag HB, Loomba R (2021). Global epidemiology of NAFLD-related HCC: trends, predictions, risk factors and prevention. Nat Rev Gastroenterol Hepatol.

[3] Masuoka HC, Chalasani N (2013). Nonalcoholic fatty liver disease: an emerging threat to obese and diabetic individuals. Ann Ny Acad Sci.

[4] Michelotti A, de Scordilli M, Palmero L (2021). NAFLD-related hepatocarcinoma: the malignant side of metabolic syndrome. Cells-Basel.

[5] Zhang C, Yang M (2021). The emerging factors and treatment options for NAFLD-related hepatocellular carcinoma. Cancers.

[6] Juanola O, Martínez-López S, Francés R (2021). Non-alcoholic fatty liver disease: metabolic, genetic, epigenetic and environmental risk factors. Int J Env Res Pub He.

[7] Singal AG, El-Serag HB (2021). Rational screening approaches for HCC in NAFLD patients. J Hepatol.

[8] Lee SS, Park SH (2014). Radiologic evaluation of nonalcoholic fatty liver disease. World J Gastroentero.

[9] Starekova J, Hernando D, Pickhardt PJ (2021). Quantification of liver fat content with CT and MRI: state of the art. Radiology.

[10] Souabni M, Hammouda O, Romdhani M (2021). Benefits of daytime napping opportunity on physical and cognitive performances in physically active participants: a systematic review. Sports Med.

[11] Wang Y, Zeng Y, Zhang X (2021). Daytime napping duration is positively associated with risk of hyperuricemia in a Chinese population. J Clin Endocrinol Metab.

[12] Lin L, Lu C, Chen W (2021). Daytime napping and nighttime sleep duration with incident diabetes mellitus: a cohort study in Chinese older adults. Int J Env Res Pub He.

[13] Patterson PD, Liszka MK, Mcilvaine QS (2021). Does the evidence support brief (≤30-mins), moderate (31–60-mins), or long duration naps (61+ mins) on the night shift? A systematic review. Sleep Med Rev.

[14] Fu J, Zhang X, Moore JB (2021). Midday nap duration and hypertension among middle-aged and older Chinese adults: a nationwide retrospective cohort study. Int J Env Res Pub He.

[15] Jarvis H, Craig D, Barker R (2020). Metabolic risk factors and incident advanced liver disease in non-alcoholic fatty liver disease (NAFLD): A systematic review and meta-analysis of population-based observational studies. PLoS Med.

[16] Maier S, Wieland A, Cree-Green M (2021). Lean NAFLD: an underrecognized and challenging disorder in medicine. Rev Endocr Metab Disord.

[17] Bastien M, Poirier P, Lemieux I (2014). Overview of epidemiology and contribution of obesity to cardiovascular disease. Prog Cardiovasc Dis.

[18] Vgontzas AN, Pejovic S, Zoumakis E (2007). Daytime napping after a night of sleep loss decreases sleepiness, improves performance, and causes beneficial changes in cortisol and interleukin-6 secretion. Am J Physiol-Endoc Metab.

[19] Devine JK, Wolf JM (2016). Determinants of cortisol awakening responses to naps and nighttime sleep. Psychoneuroendocrino.

[20] Abulizi A, Camporez J, Jurczak MJ (2019). Adipose glucocorticoid action influences whole-body metabolismvia modulation of hepatic insulin action. FASEB J.

[21] Abulizi A, Camporez J, Zhang D (2019). Ectopic lipid deposition mediates insulin resistance in adipose specific 11β-hydroxysteroid dehydrogenase type 1 transgenic mice. Metabolism.

[22] Rasch B, Dodt C, Mölle M (2007). Sleep-stage-specific regulation of plasma catecholamine concentration. Psychoneuroendocrino.

[23] Smolensky MH, Hermida RC, Castriotta RJ (2007). Role of sleep-wake cycle on blood pressure circadian rhythms and hypertension. Sleep Med.

[24] Sowers JR, Whaley-Connell A, Epstein M (2009). Narrative review: the emerging clinical implications of the role of aldosterone in the metabolic syndrome and resistant hypertension. Ann Intern Med.

[25] Kang T, Boland BB, Jensen P (2020). Characterization of signaling pathways associated with pancreatic β-cell adaptive flexibility in compensation of obesity-linked diabetes in db/db mice. Mol Cell Proteomics.

[26] Jullian-Desayes I, Trzepizur W, Boursier J (2021). Obstructive sleep apnea, chronic obstructive pulmonary disease and NAFLD: an individual participant data meta-analysis. Sleep Med.

[27] Hirono H, Watanabe K, Hasegawa K (2021). Impact of continuous positive airway pressure therapy for nonalcoholic fatty liver disease in patients with obstructive sleep apnea. World J Clin Cases.

[28] Parola M, Vajro P (2016). Nocturnal hypoxia in obese-related obstructive sleep apnea as a putative trigger of oxidative stress in pediatric NAFLD progression. J Hepatol.

[29] Lucassen EA, Rother KI, Cizza G (2012). Interacting epidemics? Sleep curtailment, insulin resistance, and obesity. Ann Ny Acad Sci.

[30] McHill AW, Melanson EL, Higgins J (2014). Impact of circadian misalignment on energy metabolism during simulated nightshift work. Proc Natl Acad Sci.

[31] Picó C, Palou M, Pomar CA (2021). Leptin as a key regulator of the adipose organ. Rev Endocr Metab Disord.

[32] Gallardo N, Bonzón-Kulichenko E, Fernández-Agulló T (2007). Tissue-specific effects of central leptin on the expression of genes involved in lipid metabolism in liver and white adipose tissue. Endocrinology.

[33] Han H, Chung SI, Park HJ (2021). Obesity-induced vitamin D deficiency contributes to lung fibrosis and airway hyperresponsiveness. Am J Resp Cell Mol.

[34] Buzzetti E, Pinzani M, Tsochatzis EA (2016). The multiple-hit pathogenesis of non-alcoholic fatty liver disease (NAFLD). Metabolism.

[35] Souza CO, Teixeira AA, Biondo LA (2017). Palmitoleic acid reduces the inflammation in LPS-stimulated macrophages by inhibition of NFκB, independently of PPARs. Clin Exp Pharmacol Physiol.

[36] Kanwal F, Kramer JR, Mapakshi S (2018). Risk of hepatocellular cancer in patients with non-alcoholic fatty liver disease. Gastroenterology.

