# Correction: Positive association of nap duration with risk of non-alcoholic fatty liver disease in an occupational population in Guangdong Province, China: a cross-sectional study

**DOI:** 10.1186/s12876-022-02292-z

**Published:** 2022-05-12

**Authors:** Chang Hong, Chengkai Wu, Pengcheng Ma, Hao Cui, Liya Chen, Ruining Li, Qimei Li, Lin Zeng, Shengwu Liao, Lushan Xiao, Li Liu, Wenyuan Li

**Affiliations:** 1 Big Data Center, Nanfang Hospital, Southern Medical University, Guangzhou, 510515 China; 2 Department of Infectious Diseases, Nanfang Hospital, Southern Medical University, Guangzhou, 510515 China; 3 Department of Radiation Oncology, Nanfang Hospital, Southern Medical University, Guangzhou, 510515 China; 4 Department of Medical Quality Management, Nanfang Hospital, Southern Medical University, Guangzhou, 510515 China; 5 Hospital Office, Nanfang Hospital, Southern Medical University, Guangzhou, 510515 China

## Correction to: BMC Gastroenterology (2022) 22:185 https://doi.org/10.1186/s12876-022-02246-5

After publication of this article [1], the authors reported that the symbol (†) indicating that Chengkai Wu and Pengcheng Ma contributed equally as first authors, is missing.

The original article [1] has been updated.
