# Supplementary material for: Positive association of nap duration with risk of non-alcoholic fatty liver disease in an occupational population in Guangdong Province, China: a cross-sectional study
Source: BMC Gastroenterol. 2022 Apr 12;22:185. doi: 10.1186/s12876-022-02246-5 (PMC9004137; doi:10.1186/s12876-022-02246-5)
Supplement: Supplementary file 1 — Additional file 1. Adjusted odds ratios (95% CI) for risk of non-alcoholic fatty liver disease using logistic regression. The table showed the odds ratios (95% CI) for risk of non-alcoholic fatty liver disease in the univariate and multivariate logistic regression models. And age, BMI, hypertension, sleeping pills using and napping over 60min per day is probably the independent risk for developing NAFLD. [file 12876_2022_2246_MOESM1_ESM.docx]

New Supplemental Table 1. Adjusted odds ratios (95% CI) for risk of non-alcoholic fatty liver disease using logistic regression.

|  | Univariate | |  | Multivariate | |
| --- | --- | --- | --- | --- | --- |
| Variable | *P* value | OR(95%CI) |  | *P* value | OR(95%CI) |
| Age,year |  |  |  |  |  |
| ≤30 | ＜0.001 | 1(ref) |  | **<0.001** |  |
| 30-40 | 0.026 | 1.632(1.061-2.511) |  | 0.016 | 1.729(1.106-2.703) |
| 40-50 | ＜0.001 | 2.408(1.474-3.931) |  | **0.001** | 2.510(1.474-4.273) |
| ＞50 | ＜0.001 | 4.660(2.574-8.439) |  | **<0.001** | 4.301(2.131-8.681) |
| Male vs Female, n (%) | 0.585 | 0.908(0.641-1.285) |  | 0.767 | 0.938(0.616-1.430) |
| BMI, kg/m2 |  |  |  |  |  |
| ＜18.5 | 0.039 | 1.669(1.026-2.714) |  | **0.009** | 1.975(1.185-3.292) |
| 18.5-23.9 | 0.102 | 1(ref) |  | **0.014** |  |
| ≥24 | 0.996 | 1.001(0.675-1.484) |  | 0.334 | 0.808(0.521-1.255) |
| Education level, n(%) | |  |  |  |  |
| High school or below | 0.087 | 1(ref) |  | 0.408 |  |
| College degree | 0.607 | 0.895(0.586-1.336) |  | 0.965 | 1.011(0.633-1.613) |
| Bachelor degree or above | 0.041 | 0.574(0.337-0.976) |  | 0.315 | 0.742(0.414-1.329) |
| Annual family personal income(yuan/person) | | |  |  |  |
| ＜20,000 | 0.725 | 1(ref) |  | 0.744 |  |
| 20-40 thousand | 0.856 | 1.075(0.494-2.338) |  | 0.660 | 1.196(0.539-2.657) |
| 40-60 thousand | 0.732 | 1.135(0.551-2.336) |  | 0.767 | 1.119(0.533-2.350) |
| 60-80 thousand | 0.360 | 0.715(0.348-1.467) |  | 0.504 | 0.778(0.372-1.626) |
| ≥80,000 | 0.718 | 0.901(0.513-1.584) |  | 0.664 | 0.878(0.486-1.583) |
| Physical activity≥1 time/week , n (%) | 0.050 | 1.404(1.000-1.972) |  | 0.129 | 1.323(0.922-1.899) |
| Hypertension, n (%) | 0.004 | 2.991(1.406-6.367) |  | 0.067 | 2.163(0.948-4.939) |
| Diabetes | 0.999 | - |  | 0.999 | - |
| Coronary disease | 0.999 | - |  | 0.998 | - |
| Sleeping pills using, n(%) | 0.010 | 2.026(1.180-3.478) |  | **0.025** | 1.891(1.082-3.306) |
| Nocturnal sleep duration＞7h, n(%) | 0.098 | 0.724(0.494-1.061) |  | 0.267 | 0.796(0.533-1.190) |
| Smoking,n(%) |  |  |  |  |  |
| never | 0.179 | 1(ref) |  | 0.281 |  |
| current | 0.060 | 1.848(0.975-3.500) |  | 0.104 | 1.806(0.886-3.680) |
| quitted | 0.593 | 1.480(0.351-6.245) |  | 0.676 | 1.371(0.312-6.028) |
| passive | 0.311 | 0.359(0.049-2.606) |  | 0.321 | 0.363(0.049-2.683) |
| Often staying up late | 0.377 | 0.859(0.612-1.204) |  | 0.299 | 0.826(0.576-1.185) |
| Daily sit-in duration |  |  |  |  |  |
| ＜2 | 0.393 | 1(ref) |  | 0.205 |  |
| 2-4 | 0.410 | 1.344(0.665-2.719) |  | 0.473 | 1.301(0.634-2.673) |
| 4-8 | 0.177 | 1.536(0.824-2.865) |  | 0.257 | 1.469(0.763-2.750) |
| ＞8 | 0.102 | 1.741(0.896-3.384) |  | 0.054 | 1.969(0.990-3.917) |
| Daytime napping duration | |  |  |  |  |
| 0 | 0.121 | 1(ref) |  | 0.089 |  |
| 1-29min | 0.478 | 1.381(0.566-3.373) |  | 0.668 | 1.221(0.492-3.031) |
| 30-59min | 0.040 | 2.076(1.070-2.898) |  | 0.066 | 1.959(0.956-4.013) |
| ≥60min | 0.042 | 2.138(1.053-3.124) |  | **0.035** | 2.253(1.061-4.786) |
| Total cholesterol | 0.026 | 1.214(1.023-1.440) |  | 0.694 | 0.849(0.376-1.916) |
| Triglycerides | 0.667 | 1.034(0.887-1.206) |  | 0.546 | 1.085(0.833-1.414) |
| HDL-C | 0.015 | 1.994(1.142-3.483) |  | 0.116 | 2.430(0.802-7.362) |
| LDL-C | 0.113 | 1.194(0.959-1.487) |  | 0.685 | 1.209(0.483-3.030) |

**Additional file 1**

File format:

Title of data: Adjusted odds ratios (95% CI) for risk of non-alcoholic fatty liver disease using logistic regression.

Description of data: The table showed the odds ratios (95% CI) for risk of non-alcoholic fatty liver disease in the univariate and multivariate logistic regression models. And age, BMI, hypertension, sleeping pills using and napping over 60min per day is probably the independent risk for developing NAFLD.
